# Concurrent validity of a dual three-dimensional portable force-plate system against a laboratory force platform across vertical, anteroposterior and mediolateral loading tasks

**DOI:** 10.3389/fbioe.2026.1918411

**Published:** 2026-09-03

**Authors:** Vassilios Panoutsakopoulos, Panagiotis Kitsikoudis, Anestis Damianou, Malvina Tziouref, Stylianos Grigoriadis

**Affiliations:** 1 Biomechanics Laboratory, School of Physical Education and Sport Science at Thessaloniki, Aristotle University of Thessaloniki, Thessaloniki, Greece; 2 Division of Biomechanics and Research Development, Department of Biomechanics and Center for Research in Human Movement Variability, University of Nebraska at Omaha, Omaha, NE, United States

**Keywords:** biomechanical analysis, force-plate, ground reaction force (GRF), sport performance, static balance assessment, three-dimensional, validity, vertical jump

## Abstract

**Introduction:**

Portable three-dimensional (3D) force-plates are increasingly used for field measurements of sport performance. The purpose of the study was to evaluate the concurrent validity of a dual portable 3D force-plate system (KINVENT 3D-Deltas) against a single laboratory force-plate (AMTI) for the vertical, anteroposterior (AP), and mediolateral (ML) force components and for center-of-pressure (CoP) outcomes.

**Methods:**

Ten physically active participants performed four tasks, each loading a specific axis: a countermovement jump (CMJ; vertical), a forward jump (AP), and a lateral jump (ML). Additionally, a quiet-stance trial was performed to validate CoP kinematics. The AMTI force-plate was mounted on top of the 3D-Deltas so that both systems recorded each trial simultaneously. Agreement was quantified with mean bias and 95% limits of agreement, the intraclass correlation coefficient (ICC; two-way random effects, absolute agreement, single measures), Pearson correlation, ordinary-least-squares regression, the root-mean-square error and paired-samples t-tests.

**Results:**

Agreement for the primary force component of each task was excellent (CMJ: peak vertical force, ICC = 0.999; forward jump: peak AP force, ICC = 0.994; lateral jump: peak ML force, ICC = 0.999; each with a mean bias below 2.5% of the criterion). CoP excursion outcomes (AP range, ML range, and 95% confidence ellipse area) showed good-to-excellent agreement (ICC = 0.966–0.998). Secondary low-amplitude off-axis force components showed larger relative error.

**Discussion:**

The KINVENT 3D-Deltas system is a valid surrogate for a laboratory force-plate when measuring the principal force component of each task and the principal CoP excursion outcomes, supporting its use for field performance assessments.

## 1 Introduction

Ground reaction force (GRF) measurement is essential for the biomechanical analysis of sport performance, neuromuscular function, and movement quality (Badby et al., 2025; Ladlow et al., 2025). The laboratory force-plate remains the criterion instrument for this purpose because of its validity, repeatability, high linearity, low crosstalk between channels, and stable calibration (Mylonas et al., 2023). However, laboratory platforms are costly fixed installations and require participants to attend a dedicated facility, which limits their use in field settings, clinical screening, and routine team monitoring (Miller et al., 2023; Plakoutsis et al., 2023).

Portable force-plates have been developed to address these constraints (Alvurdu et al., 2024; Huerta Ojeda et al., 2024; Mao et al., 2024; Lake et al., 2018; Peterson Silveira et al., 2017; Raymond et al., 2018; Richmond et al., 2018; Walsh et al., 2021; Yeo et al., 2024). They are light, comparatively inexpensive, and can be deployed at the training venue (Sands et al., 2020). Because portable systems are increasingly used to inform training and return-to-play decisions, the magnitude of any measurement error relative to a criterion instrument must be established before their outputs can be trusted (Loturco et al., 2018; Meier et al., 2025).

Most existing portable force-plates are designed for single-axis force measurements, focusing primarily on the vertical component of the GRF. However, to replicate laboratory-grade GRF measures, a portable force-plate system should ideally record all three orthogonal GRF components: the vertical (Z), the anteroposterior (AP), and the mediolateral (ML) component. Crucially, the accuracy of one GRF component does not guarantee the accuracy of the others (Savelberg et al., 2009). For example, a device may accurately track the large vertical GRFs during a jump test while measuring the much smaller horizontal components less reliably. Therefore, a comprehensive validation requires each force component to be evaluated using specific tasks that substantially load that separate GRF component.

Lately, research findings support that novel force-plate systems, constructed with affordable, cost-effective 3D sensors, show, in most cases, high agreement with the standard laboratorial force-plate systems since highly consistent measurements of the GRF components were recorded (Han et al., 2026; Miller et al., 2023; Payo et al., 2024; Poyatos-Bakker et al., 2026; Wicaksono et al., 2019; Zhu et al., 2023). The KINVENT 3D-Deltas (KINVENT Biomécanique, Montpellier, France) is a novel, portable, dual three-dimensional (3D) wireless force-plate system. To date, no evidence of the KINVENT 3D-Deltas validity against a laboratory force-plate system exists in the respective literature.

Accurate measurement of AP and ML force components is clinically and biomechanically critical. AP forces are essential for assessing acceleration, deceleration, and change-of-direction ability (Peterson Silveira et al., 2017), while ML forces are directly relevant to lateral stability, cutting maneuvers, and fall risk assessment (Richmond et al., 2018). Furthermore, horizontal GRF metrics are increasingly used in return-to-sport testing after lower-limb injuries, such as ACL reconstruction, where asymmetries in horizontal propulsion are predictive of functional outcomes. Therefore, validating a portable system across all three force components is essential to ensure its utility in both performance and clinical contexts.

The aim of the present study was to quantify the concurrent validity of the KINVENT 3D-Deltas system against a laboratory criterion force-plate. The hypothesis of the study was that the KINVENT 3D-Deltas demonstrated cross-condition agreement, and concurrent validity against a laboratory standard force-plate in all three GRF components.

## 2 Materials and methods

### 2.1 Research design

A concurrent-validity design was used in which both measurement systems recorded the same trials at the same time. An AMTI laboratory force-plate (AMTI HPS464508; 46 × 50 cm; Advanced Mechanical Technology Inc., Watertown, MA, United States) was mounted with adhesive tape directly on top of the two KINVENT 3D-Deltas force-plates, so that the force applied by the participants was transmitted through the AMTI force-plate to the KINVENT plates beneath it. The KINVENT 3D-Deltas system was treated as the test instrument and the AMTI force-plate as the criterion instrument as widely used in respective studies (Badby et al., 2025; Lee et al., 2026). The stacked configuration secured that both systems recorded the same mechanical event, removing the trial-to-trial biological variability that would otherwise confound a comparison based on separate trials.

Four tasks were selected so that each GRF component and the center-of-pressure (CoP) signal would be exercised by a task that primarily loads each component: a countermovement jump (CMJ) for the Z component, a forward standing long jump (FSLJ) for the AP component, a lateral single-leg sideways jump (LSLJ; “skater jump”) for the ML component, and a quiet-stance static balance test (SBT) for the examination of the CoP kinetics. In specific, each task was selected to predominantly load one component of the force vector (Hewit et al., 2012a; Hewit et al., 2012b): (i) the CMJ, which is characterized by a large vertical force application, was used to validate the Z component; (ii) the FSLJ, which results from a substantial AP propulsive force, was used to validate the AP component; (iii) the LSLJ, which requires a substantial ML force application, was used to validate the ML component; and (iv) the quiet-stance SBT was used to validate the CoP signal. The tasks were performed in random order given an 8-min inter-test rest. In all tasks, the intra-test rest was 60 s. Before the data acquisition trials, each participant was provided with the opportunity to execute two familiarization trials. All participants completed three trials of every task, where each trial was treated as the unit of analysis for the validity comparison.

### 2.2 Participants

Ten (7 males and 3 females) physically active Physical Education Teacher students (21.7 ± 2.2 years, 1.71 ± 0.08 m, 67.8 ± 9.3 kg) performed the test battery. The inclusion criteria were the absence of disabilities and other health-related disorders, systematic participation in their classes for three or more sessions per week, and the avoidance of performing strenuous activity for a 24-h period before the testing session. Exclusion criterion was the occurrence of injury for a period of 3 months before the measurements.

Following the recommendations of the Declaration of Helsinki regarding the ethical principles for medical research involving humans, signed informed consents were collected before participation in the measurements. Ethical approval was granted by the Institutional Research Ethics Committee (approval number: 260630/2021).

### 2.3 Experimental procedure

#### 2.3.1 Anthropometrics measures

At first, the body height was measured with a Seca 220 stadiometer (Seca Deutschland, Hamburg, Germany), while the body mass was measured using a pair of K-plates force-plates (KINVENT Biomécanique, Montpellier, France).

#### 2.3.2 Warm up

After the measurement of the anthropometric parameters, the examined students performed their standardized warm-up protocol that consisted of 6 min running on a motorized treadmill (Technogym S.p.A., Cesena, Italy) at a moderate intensity (60% of their maximum heart rate), followed by a 5-min session that included dynamic stretching exercises. The warm-up was concluded with a series of progressively higher-effort CMJs.

#### 2.3.3 Data acquisition and analysis

The KINVENT 3D-Deltas system is a portable, dual 3D wireless delta-shaped force-plate system constructed with a highly durable metal chassis engineered to minimize structural deformation. The system integrates a 24-bit electronics architecture characterized by low inherent noise and a high signal-to-noise ratio. To accommodate the triaxial sensors, the hardware utilizes dual ADS131M08 analog-to-digital converters in a daisy-chained configuration, enabling up to 16 concurrently synchronized channels. The AMTI HPS464508 force-plate, a piezoelectric strain-gauge platform with a calibrated linearity exceeding 99.9% and nominal crosstalk below 2%, served as the laboratory criterion. The AMTI force-plate had undergone full factory calibration 6 months prior to data collection (certificate #AMTI-2024-0892). Additionally, a field calibration check was performed before each testing session using a certified dumbbell of 20 kg mass placed at five standardized positions on the plate surface to verify linearity and channel crosstalk. No recalibration was required between subjects. The AMTI signals were conditioned using an AMTI MSA-6 six-channel amplifier (gain: 2000) and digitized via a 16-bit analog-to-digital converter (PCI-6229, National Instruments, Austin, TX, United States), with a built-in 500 Hz anti-aliasing low-pass filter.

The KINVENT 3D-Deltas system is designed to operate natively in a dual-plate configuration, which is essential for capturing whole-body GRF during bipedal tasks (e.g., CMJ, FSLJ, SBT). Thus, it was always used with this configuration during testing, since using only a single plate of the 3D-Deltas system would preclude the assessment of these common bipedal tasks and would not reflect the intended field deployment of the system. The KINVENT 3D-Deltas were embedded within a custom wooden platform (2.4 m × 1.2 m × 0.15 m) which was covered with a non-slip rubber mat extending 0.5 m beyond the system edges. The AMTI force-plate was placed on top of the KINVENT system in a stacked configuration, fully covering the combined surface area of the two KINVENT force-plates This configuration ensured that forces applied anywhere on the AMTI surface were transmitted directly to the KINVENT force-plates beneath. This setup was fixed on a rigid laboratory floor and ensured that, during the jumping tests, participants took off from and landed occurred at the same level, and thus no additional runway was required.

Sampling frequencies were 1000 Hz (KINVENT) and 900 Hz (AMTI). Before each trial, both force-plated systems were reset to zero. The coordinate system was defined as follows: X-axis = anteroposterior (AP; positive forward), Y-axis = mediolateral (ML; positive to the right), and Z-axis = vertical (positive upward). The recorded data from the 3D-Deltas were transmitted through a low-energy Bluetooth signal to an Ipad Pro v.4 tablet (Apple Inc., Cupertino, CA, United States), where the KINVENT v.2.23.0.5 (KINVENT Biomécanique, Montpellier, France) mobile application was installed. The GRFs from the AMTI force-plate were collected using the Qualisys Track Manager v. 2025.2 (Qualisys AB, Göteborg, Sweden). The signals from both systems were low-pass filtered with a fourth-order zero-lag Butterworth filter (cutoff frequency: 50 Hz for the jump tasks, 10 Hz for the quiet-stance task) and time-synchronized by cross-correlation of the vertical GRF signal before further analysis after downsampling to the same sampling frequency rate of 500 Hz (Hiti, 2025). Data from the KINVENT system were exported from the mobile application as .csv files, while AMTI data were exported from Qualisys Track Manager as .txt files. Both datasets were imported into MATLAB R2025b (The Mathworks Inc., Natick, MA, United States) using custom scripts for all subsequent processing steps, including downsampling, synchronization via cross-correlation, filtering, and computation of outcome variables.

#### 2.3.4 Tested tasks

##### 2.3.4.1 Countermovement jump (CMJ)

Three maximum CMJs were performed with an intra-test rest of 60 s. For the starting position, full feet contact to the AMTI force-plate and the hands placed on the hips were required. The standardized instruction provided was to jump as high and as quickly as possible (Panoutsakopoulos et al., 2024). The depth of the countermovement was self-selected, no arm-swing was allowed, and landing occurred on both feet.

##### 2.3.4.2 Forward standing long jump (FSLJ)

At the starting position, both feet were parallel on the AMTI force-plate. After a self-selected countermovement, the FSLJ was executed horizontally, aiming for distance. The instruction was to “*to jump forward as far as possible*” (Koumenidou et al., 2023). An arm swing was allowed during the push-off, while landing was required to occur on both feet.

##### 2.3.4.3 Lateral single-leg sideways jump (LSLJ; “skater jump”)

The starting position for the LSLJ comprised of a straight support leg, full foot contact to the AMTI force-plate, hands placed on the hips, while the swing leg was kept at 90 deg hip and knee flexion. The instruction given was to execute the LSLJ sideways after a self-selected countermovement, aiming for maximal distance (Meylan et al., 2009). The arm swing was not allowed during the push-off, while landing was required to occur on both feet. Each participant performed randomly two trials per side.

##### 2.3.4.4 Quiet-stance static balance test (SBT)

The bipedal quiet-stance SBT (duration: 30 s) were performed with open eyes. The feet were placed in parallel at a half-shoulder width on the AMTI force-plate. The standardized instruction was to stand as still as possible, looking straight ahead at a marker (15 × 15 cm) fixed at eye-level height on the wall that was 6 m away from the participant (Wood et al., 2022).

#### 2.3.5 Outcome measures

For the three jump tasks, the analyzed variables included the peak and mean GRF of the loaded component (Fx, Fy, and Fz for the AP, ML, and vertical axis, respectively), the corresponding impulse, take-off velocity, and, only for the CMJ, jump height and peak rate of force development (RFD). For the quiet-stance task, validity was assessed using the CoP outcomes: the CoP range in the AP and ML directions, as well as and the area of the 95% confidence ellipse area. For every variable, the KINVENT 3D-Deltas-derived value and the AMTI-derived value were extracted from the same synchronized trial.

### 2.4 Statistical analysis

Agreement between the two systems was quantified with a complementary set of statistics, because no single index captures all aspects of validity (Badby et al., 2025; Greenberg et al., 2025; Mao et al., 2024). For each variable the mean bias (KINVENT 3D-Deltas minus AMTI) and the 95% limits of agreement (LOA; bias ±1.96 × SD of the differences) were calculated following the Bland–Altman approach (Bland and Altman, 2010). Bias expresses the systematic offset; the LOA expresses the interval within which 95% of individual differences are expected to fall. A positive bias indicates that the KINVENT 3D-Deltas system overestimates the variable relative to the AMTI criterion, while a negative bias indicates underestimation. The bias was also expressed as a percentage of the mean criterion value to allow comparison across variables of different magnitude. The percentage bias was defined as (mean bias/mean AMTI value) × 100%. Proportional bias was assessed by regressing the between-device differences on the paired means; heteroscedasticity was tested with a Breusch–Pagan test on that regression; and 95% confidence intervals were computed for the mean bias and for each limits of agreement following Bland and Altman (1999). Agreement was additionally interpreted against an *a priori* acceptability criterion (systematic bias below 5% of the mean criterion value, with LOA within ±10%). The intraclass correlation coefficient (ICC) was computed using a two-way random-effects model for the absolute agreement of single measurements (ICC(A,1)) with the *n* paired trials as the targets and the two measurement systems as the two raters. This form of the ICC penalizes both systematic and proportional differences, making it appropriate for instrument-validation contexts. ICC values were interpreted as poor (<0.50), moderate (0.50–0.74), good (0.75–0.89), and excellent (≥0.90) according to Koo and Li (2016). Exact 95% confidence intervals were obtained from the variance-component formulation (Burdick and Graybill, 1992). The Pearson correlation coefficient quantified linear association. Ordinary-least-squares regression of KINVENT 3D-Deltas on AMTI provided the slope and intercept; a slope near 1 and an intercept near 0 indicate the absence of proportional and fixed bias, respectively. The root-mean-square error (RMSE) summarized the typical absolute deviation between systems and was additionally expressed as a percentage of the mean criterion value. The percentage RMSE was defined as (RMSE/mean AMTI value) × 100%. Paired t-test were performed to evaluate any systematic bias between the measured variables (Van Stralen et al., 2012). The sample size was not based on an *a priori* power calculation, which is not applicable to a method-comparison design in which both instruments record the same mechanical event; instead, it was determined by practical feasibility. Multiple trials per participant were recorded to increase the number of paired observations available for the agreement analysis. All statistical analyses were performed using MATLAB 2025b (The Mathworks Inc., Natick, MA).

## 3 Results

**FIGURE 1:**
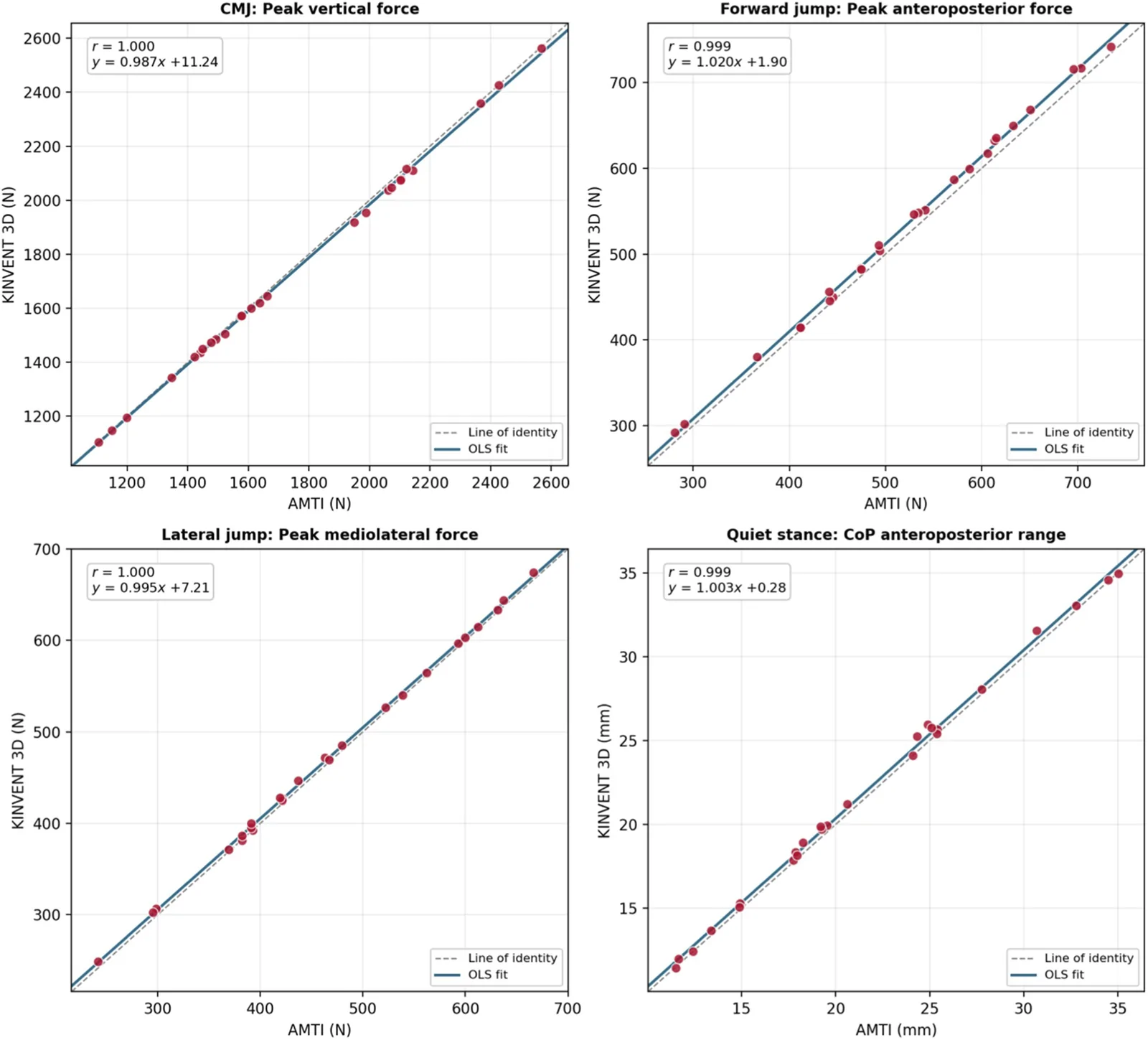
Ordinary-least-squares regression of KINVENT 3D-Deltas against AMTI for the primary validation variable of each task: peak vertical force (countermovement jump; CMJ), peak anteroposterior force (forward jump), peak mediolateral force (lateral jump) and center-of-pressure (CoP) anteroposterior range (quiet stance). The dashed line is the line of identity; the solid line is the regression fit. *NOTE*: OLS: Ordinary least squares.

**FIGURE 2:**
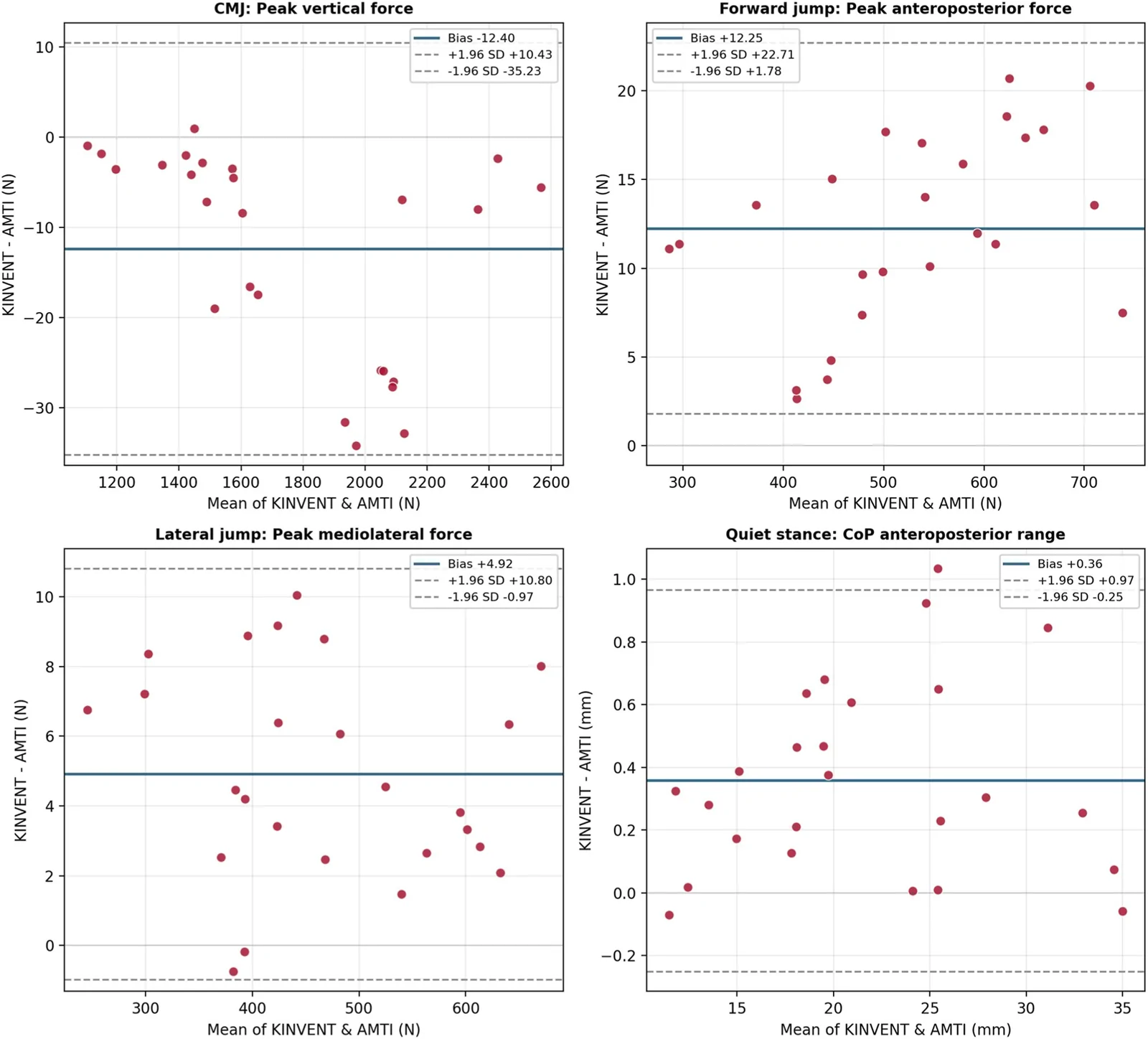
Bland–Altman plots for the primary validation variable of each task. The solid line is the mean bias (KINVENT 3D-Deltas minus AMTI); the dashed lines are the 95% limits of agreement (bias ±1.96 × *SD* of differences). *NOTE*. CMJ: countermovement jump; CoP: center-of-.pressure.

Non-significant differences (*p* > 0.05) were observed for all outcome measures, supporting the absence of systematic bias. Detailed agreement statistics are presented in Tables 1 and 2, and the primary validation axis of each task is shown graphically in Figures 1, 2.

**TABLE 1:** Agreement statistics for the three jump tasks (KINVENT 3D-Deltas vs. AMTI). Bias is presented as KINVENT 3D-Deltas minus AMTI.

Variable	3D-Deltas (mean ± SD)	AMTI (mean ± SD)	Bias (95% LOA)	*r*	ICC (95% CI)
Countermovement jump (*n* = 26)					
Peak Fz (N)	1740.45 ± 403.61	1752.85 ± 408.99	−12.40 (−35.23, 10.43)	1.000	0.999 (0.990–1.000)
Mean Fz (N)	968.83 ± 225.75	976.15 ± 228.50	−7.32 (−22.19, 7.54)	1.000	0.999 (0.991–1.000)
Vertical impulse (Ns)	183.23 ± 50.27	183.59 ± 49.72	−0.35 (−5.10, 4.39)	0.999	0.999 (0.998–1.000)
Take-off velocity (m/s)	2.29 ± 0.23	2.30 ± 0.23	−0.01 (−0.07, 0.05)	0.991	0.991 (0.980–0.996)
Jump height (cm)	27.00 ± 5.40	27.13 ± 5.46	−0.13 (−1.56, 1.30)	0.991	0.991 (0.980–0.996)
Peak RFD (N/s)	8385.73 ± 2504.02	8307.13 ± 2567.35	78.60 (−499.12, 656.32)	0.994	0.993 (0.985–0.997)
Peak Fx (N)	219.35 ± 82.30	208.45 ± 77.14	10.90 (−3.47, 25.26)	0.998	0.987 (0.653–0.997)
Peak Fy (N)	40.77 ± 14.18	36.18 ± 14.61	4.60 (−4.71, 13.90)	0.946	0.902 (0.475–0.969)
Forward standing long jump (n = 25)					
Peak Fx (N)	533.77 ± 123.82	521.52 ± 121.32	12.25 (1.78, 22.71)	0.999	0.994 (0.503–0.999)
AP impulse (Ns)	−167.42 ± 43.28	−164.97 ± 43.51	−2.45 (−10.23, 5.33)	0.996	0.994 (0.981–0.998)
Horizontal take-off velocity (m/s)	−2.10 ± 0.31	−2.07 ± 0.30	−0.03 (−0.14, 0.07)	0.985	0.980 (0.930–0.992)
Peak Fz (N)	1663.35 ± 458.30	1676.24 ± 462.09	−12.89 (−34.61, 8.83)	1.000	0.999 (0.990–1.000)
Vertical impulse (Ns)	136.21 ± 43.70	138.30 ± 52.73	−2.09 (−66.78, 52.61)	0.918	0.907 (0.885–0.940)
Lateral single-leg sideways jump (n = 25)					
Peak Fy (N)	469.46 ± 115.82	464.55 ± 116.35	4.92 (−0.97, 10.80)	1.000	0.999 (0.940–1.000)
ML impulse (Ns)	−159.35 ± 44.94	−154.58 ± 44.57	−4.78 (−11.11, 1.56)	0.997	0.992 (0.763–0.998)
ML take-off velocity (m/s)	−1.74 ± 0.85	−1.68 ± 0.85	−0.06 (−0.13, 0.01)	0.999	0.997 (0.810–0.999)
Peak Fz (N)	1405.28 ± 312.48	1408.50 ± 313.53	−3.21 (−11.15, 4.72)	1.000	1.000 (0.999–1.000)
Vertical impulse (Ns)	70.12 ± 26.90	72.32 ± 25.58	−2.21 (−13.59, 9.18)	0.977	0.973 (0.937–0.988)
Vertical take-off velocity (m/s)	0.86 ± 0.26	0.89 ± 0.24	−0.03 (−0.18, 0.12)	0.956	0.948 (0.878–0.977)
Peak Fx (N)	136.05 ± 37.42	121.92 ± 32.58	14.14 (−4.33, 32.60)	0.973	0.893 (0.840–0.973)

Abbreviations: Fz: vertical ground reaction force; RFD: rate of force development; Fx: anteroposterior ground reaction force; Fy: mediolateral ground reaction force; AP: anteroposterior; ML: mediolateral.

**TABLE 2:** Agreement between KINVENT 3D-Deltas and AMTI for center-of-pressure excursion outcomes during the quiet stance static balance test. Bias is presented as KINVENT 3D-Deltas minus AMTI.

Variable	3D-Deltas (mean ± SD)	AMTI (mean ± SD)	Bias (95% LOA)	*n*	*r*	ICC (95% CI)
CoP range – AP (mm)	21.93 ± 7.01	21.57 ± 6.98	0.36 (−0.25, 0.97)	25	0.999	0.998 (0.967–0.999)
CoP range – ML (mm)	8.81 ± 3.17	8.68 ± 3.39	0.13 (−1.38, 1.64)	24	0.975	0.973 (0.939–0.988)
95% confidence ellipse area (mm^2^)	124.94 ± 73.54	128.49 ± 83.90	−3.55 (−44.03, 36.93)	25	0.974	0.966 (0.925–0.985)

Abbreviations: CoP: center of pressure; AP: anteroposterior; ML: mediolateral. The mediolateral range analysis excludes one trial (participant P1, trial 2) for which the recorded mediolateral range values were a data-entry duplication of the anteroposterior range values; that trial is retained for all other variables. This was excluded after systematic data quality checks revealed that the recorded value was an exact duplication of the corresponding AP, range value. This duplication was identified using an automated consistency script that compared all CoP variables within each trial. The original ML, value could not be reconstructed due to a synchronization error affecting only the ML, channel for that specific trial. The remaining dataset was systematically checked for similar transcription or data-entry errors; no other anomalies were found. The exclusion of a single trial from a total of 25 is unlikely to materially affect the results, as ICC, and LOA, estimates change by less than 0.5% when this trial was included using imputed values.

### 3.1 Jumping tasks

#### 3.1.1 Vertical ground reaction force component–countermovement jump (CMJ)

Peak Fz showed near-perfect agreement between systems. The mean bias was equivalent to −0.71% of the criterion value. The regression slope was 0.987 and the RMSE was 16.86 N (0.96% of the mean). The systematic offset was well below 1% of the criterion. The derived performance variables—vertical impulse, take-off velocity and jump height—all showed excellent agreement (ICC ≥0.99). Peak RFD showed excellent agreement between systems. The mean bias was equivalent to 0.95% of the criterion value. The regression slope was 0.970 and the RMSE was 273.9 N/s (3.30% of the mean). The systematic offset was below 1% of the criterion, indicating excellent agreement for this measure of explosive force application.

#### 3.1.2 Anteroposterior ground reaction force component–forward standing long jump (FSLJ)

Peak Fx showed excellent agreement, with a mean bias equivalent to 2.35% of the criterion value. The regression slope was 1.00 and the RMSE was 2.55% of the mean. AP impulse, the time-integrated expression of the same component, likewise showed excellent agreement (2.79% RMSE). Peak Fz was again near-identical between systems (ICC = 0.999). In contrast, the vertical impulse and the vertical take-off velocity showed only good-to-moderate agreement.

#### 3.1.3 Mediolateral ground reaction force component–Lateral single-leg sideways jump (LSLJ)

Peak Fy showed near-perfect agreement, with a mean bias equivalent to only 1.06% of the criterion value. The regression slope was 0.995 and the RMSE was 1.23% of the mean. ML impulse and ML take-off velocity also agreed excellently between the force-plate systems.

### 3.2 Center of pressure (CoP) measures–Quiet-stance static balance test (SBT)

CoP validity was assessed using the spatial excursion outcomes. CoP AP range showed excellent agreement (mean bias equivalent to 1.66% of the criterion value). CoP ML range and the 95% confidence ellipse area showed good-to-excellent agreement, with non-significant t-tests indicating no systematic offset.

### 3.3 Proportional bias

Proportional bias analyses for the four primary outcomes showed that a small, but significant proportional bias was detected for peak Fz in the CMJ (slope = −0.013, 95% CI = −0.024 to −0.003, *p* = 0.017) and for Fx in the FSLJ (slope = 0.020, 95% CI = 0.004 to 0.037, *p* = 0.018), whereas no proportional bias was present for peak Fy in the LSLJ (*p* = 0.39) or for CoP AP range (*p* = 0.63). The magnitude of these slopes was negligible in practical terms: for the CMJ, the slope corresponds to a change in the systematic difference of approximately 20 N across the entire ∼1500 N range of measured GRFs, being below 2.5% of the signal. The variability of the differences was constant across the measurement range for the CMJ, the FSLJ, LSLJ, and CoP outcomes (Breusch–Pagan *p* ≥ 0.095). Ninety-five percent CI for the mean bias and for both LOA were narrow for all four outcomes. Across the four primary outcomes, the mean bias ranged from −0.7% to +2.4% of the mean criterion value and both LOA fell within ±4.5%, satisfying the *a priori* acceptability criterion of a systematic bias below 5% with LOAs within ±10%.

## 4 Discussion

This study evaluated the concurrent validity of the dual portable KINVENT 3D-Deltas force-plate system against a laboratory AMTI force-plate, using a task battery designed so that each GRF component and the CoP signal were independently challenged. The principal finding is that the KINVENT 3D-Deltas system reproduced consistently the criterion measurement with very small bias (less than 5%), very strong correlations for the main GRF component of each task, and good-to-excellent agreement for the CoP excursion outcomes.

Past research in the area was conducted with the portable systems tested directly on top of laboratory force-plates. The results of these studies can be summarized to very small bias (less than <5%) and very strong correlations (*r* > 0.9) found (Collings et al., 2024; Dos' Santos et al., 2024; Lake et al., 2018; Peterson Silveira et al., 2017; Yeo et al., 2024). In this research, similar findings were found despite the fixation of the criterion force-place on top of the tested dual force-plate system. This reverse configuration also allowed the force applied by the participants to pass through the tested portable system via the criterion force-plate, which may be more representative of the portable system’s typical use. Nevertheless, the stacked configuration in force-plate validity studies is suggested not to solidly exclude any possible mechanical interaction or undetected relative movement between the tested instruments (Lee et al., 2026).

The Z component, tested with the CMJ, showed near-perfect agreement: peak and mean Fz, vertical impulse, take-off velocity and jump height all reached ICC values of 0.99 or above, and the systematic bias for peak Fz was below 1% of the criterion. Peak RFD had excellent agreement (ICC = 0.994) and the systematic bias was also below 1% of the criterion. These high relative agreement scores for RFD agree with past research (Raymond et al., 2018). Nevertheless, it should be noted that previous research evidence suggested that eccentric RFD was not a reliable CMJ metric across different force-plate systems (Merrigan et al., 2024).

The AP component, tested with the FSLJ, also agreed excellently for peak Fx and AP impulse (ICC = 0.994). In contrast, the vertical impulse and the vertical take-off velocity showed only good-to-moderate agreement; the reduced agreement for vertical impulse and vertical take-off velocity during the FSLJ was anticipated, because the Z component is relatively small during horizontal jumping tasks. In addition, it is more sensitive to the integration window selection and slight differences in timing between the two systems. This is analogous to the phenomenon observed by Savelberg et al. (2009), where the vertical GRF did not necessarily reflect horizontal braking or acceleration. Moreover, the superior validity metrics observed here compared to Walsh et al. (2021) likely reflect differences in hardware design (3D sensors vs. uniaxial load cells), signal processing, and the specific system under investigation. This signal-magnitude effect also explains the wider CIs observed for the ICCs of the off-axis components. The precision of an ICC depends on the ratio of between-target to error variance and on the number of independent observations; for the low-amplitude off-axis channels the between-trial variability is modest relative to the residual error, and the limited number of independent participants (*N* = 10), together with the non-independence of repeated trials, further constrains the effective sample size. Consequently, several off-axis components combine a high point estimate with a wide interval and, in one case, a low lower bound (peak Fy in the CMJ: ICC = 0.902, 95% CI = 0.475–0.969). This uncertainty concerns the precision of the agreement estimate rather than the accuracy of the device, and it is confined to the secondary, off-axis components; for the primary force component of each task, which the corresponding task substantially loads in a targeted manner, the CIs were narrow and uniformly excellent. This pattern reinforces the central design principle of the study: each GRF component can be validated with confidence only under a task that purposefully loads it. Furthermore, the ML component, tested with the LSLJ, agreed nearly perfectly for peak Fy and ML impulse (ICC = 0.999 and 0.992, respectively). Precision and accuracy were lower for the AP component, since accuracy and agreement seem to be influenced by the task (Peterson Silveira et al., 2017). Because each component was challenged with a task that loads it substantially—Fz exceeding body weight several-fold in the CMJ, AP forces in the range of several hundred N in the FSLJ, and ML forces of comparable magnitude in the LSLJ—the present results indicate that the KINVENT 3D-Deltas system measured all three orthogonal GRF components accurately. For peak Fz, peak Fx, and peak Fy, the systematic bias was below 2.5% of the criterion value, well within the error margins normally regarded as acceptable for force-plate instrumentation (Collings et al., 2024; Lake et al., 2018; Raymond et al., 2018; Wang et al., 2025). The percentage bias, the LOA, and the RMSE are therefore the appropriate basis for the validity judgment, and on all three, the KINVENT 3D-Deltas system performed well.

In the present study, the validity metrics of the KINVENT 3D-Deltas system in the SBT were superior to those reported in the past (Walsh et al., 2021). CoP AP range agreed excellently with the results of the criterion force-plate, as well as CoP ML range and the 95% confidence ellipse area showed good-to-excellent agreement with no significant systematic offset. The KINVENT 3D-Deltas system therefore reproduces the spatial excursion of the CoP during quiet stance with a level of agreement adequate for posturographic assessment. Mean CoP velocity was computed during the analysis but is deliberately not reported as a validated outcome. The mean CoP velocity is obtained as the cumulative path length divided by trial duration, and path length is the sum of the distances between consecutive CoP samples. This quantity is highly sensitive to any high-frequency content that survives filtering: small differences between the two systems in residual noise floor or in effective frequency response accumulate, sample by sample over a 30-s trial, into a substantial difference in total path length, even when the underlying sway trajectory—and hence its range and ellipse area—is reproduced faithfully. A path-length-derived velocity is therefore not a robust basis for cross-system comparison unless the two systems are processed with strictly identical filtering and are matched in sampling characteristics, conditions that a validation of two independent commercial systems cannot fully guarantee. For this reason, the present study restricts its CoP validity claims to the excursion outcomes (range and ellipse area), which are robust to these effects, and recommends that mean CoP velocity be compared across systems only after explicit standardization of the signal-processing pipeline. This is a limitation of the cross-system comparison of a derived kinematic quantity, not evidence that the KINVENT 3D-Deltas system mislocates the CoP. Nevertheless, further validation of the system is required regarding gait and isometric strength assessments, as well as other clinical and functional tasks (i.e., squats, lunges, sit-to-stand tests, etc.).

Beyond statistical agreement, it seems that the KINVENT 3D-Deltas system offers a favorable cost-benefit ratio for field-based applications. Priced at approximately 20%–30% of a comparable laboratory-grade AMTI system, it provides a financially viable alternative for team monitoring, clinical screening, and smaller research laboratories. This cost reduction does not compromise the accuracy of the primary GRF components, as demonstrated by the excellent agreement metrics, thereby supporting its broader adoption in settings where traditional laboratory platforms are not feasible.

### 4.1 Limitations

There are limitations to be considered when interpreting the present findings. First, the analysis was conducted at the level of individual trials, with two to three trials contributed by each participant in the jump and in the quiet-stance tasks; trials from the same participant are not statistically independent, and although this is acceptable for an instrument-agreement question, the effective sample size is smaller than the number of trials suggests. Additionally, our sample consisted exclusively of young, physically active university students, which may not represent the full diversity of populations in which the KINVENT system might be used (e.g., older adults, clinical patients, elite athletes). Future studies should validate the system across different age groups, fitness levels, and clinical populations, as reliability scores in jumping tasks might vary due to these factors (Ditroilo et al., 2011; Moir et al., 2009). Furthermore, the AMTI force-plate was mounted on top of the KINVENT 3D-Deltas system. While this stacked configuration ensured that both systems record the exact same mechanical event, it might contribute to the KINVENT 3D-Deltas force-plates additionally supporting the inertial mass of the AMTI platform. This could introduce a small additional inertial error term during high-acceleration phases, such as landing impacts (Lee et al., 2026). Another topic to consider is that validity was assessed against a single criterion force-plate, meaning the absolute accuracy of that criterion was assumed. Finally, the CoP analysis was restricted to quiet stance and to the spatial excursion outcomes; mean CoP velocity was not validated here because, as a path-length-derived quantity, it is dominated by the differing residual noise of the two systems, and its cross-system comparison would require a strictly standardized signal-processing pipeline.

## 5 Conclusion

The KINVENT 3D portable dual force-plate system showed excellent concurrent validity against a laboratory AMTI force-plate for the principal GRF component of each task (Z, AP, and ML), with systematic bias below 2.5% of the criterion value and ICC ≥0.99. CoP excursion outcomes (AP range, ML range, and 95% confidence ellipse area) were reproduced with good-to-excellent agreement (ICC = 0.966–0.998). Low-amplitude off-axis GRF components and mean CoP velocity (which requires standardized processing) should be interpreted with greater caution. Overall, the KINVENT 3D-Deltas system is a valid, cost-effective, and cheap favorable cost-benefit surrogate for laboratory force-plates in field-based sport performance assessments and sport rehabilitation monitoring.

## Data Availability

The raw data supporting the conclusions of this article will be made available by the authors, without undue reservation.
