# Supplementary material for: Concurrent validity of a dual three-dimensional portable force-plate system against a laboratory force platform across vertical, anteroposterior and mediolateral loading tasks
Source: Front Bioeng Biotechnol. 2026 Sep 3;14:1918411. doi: 10.3389/fbioe.2026.1918411 (PMC13582481; doi:10.3389/fbioe.2026.1918411)
Supplement: Supplementary file 1 [file Supplementaryfile1.pdf]

Supplementary Material

1.1 Supplementary Figures

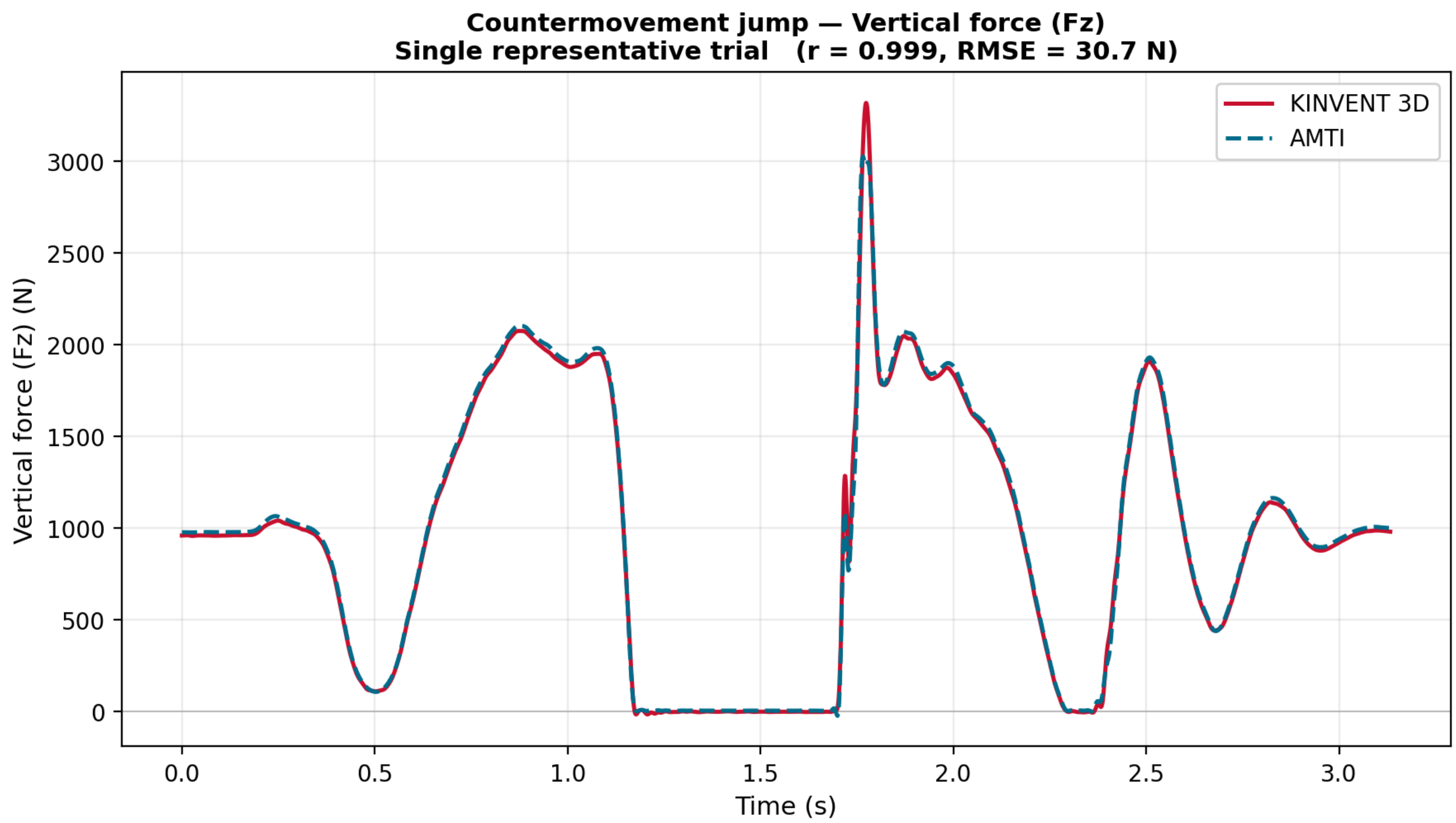

Supplementary Figure 1. Representative vertical ground reaction force curves (Fz) recorded during a vertical countermovement jump. The root-mean-square error (RMSE) for this trial is displayed above the graph. AMTI: AMTI HPS464508 force-plate (Advanced Mechanical Technology Inc., Watertown, MA, USA); KINVENT 3D: KINVENT 3D-Deltas portable dual force-plate system; KINVENT Biomécanique, Montpellier, France).

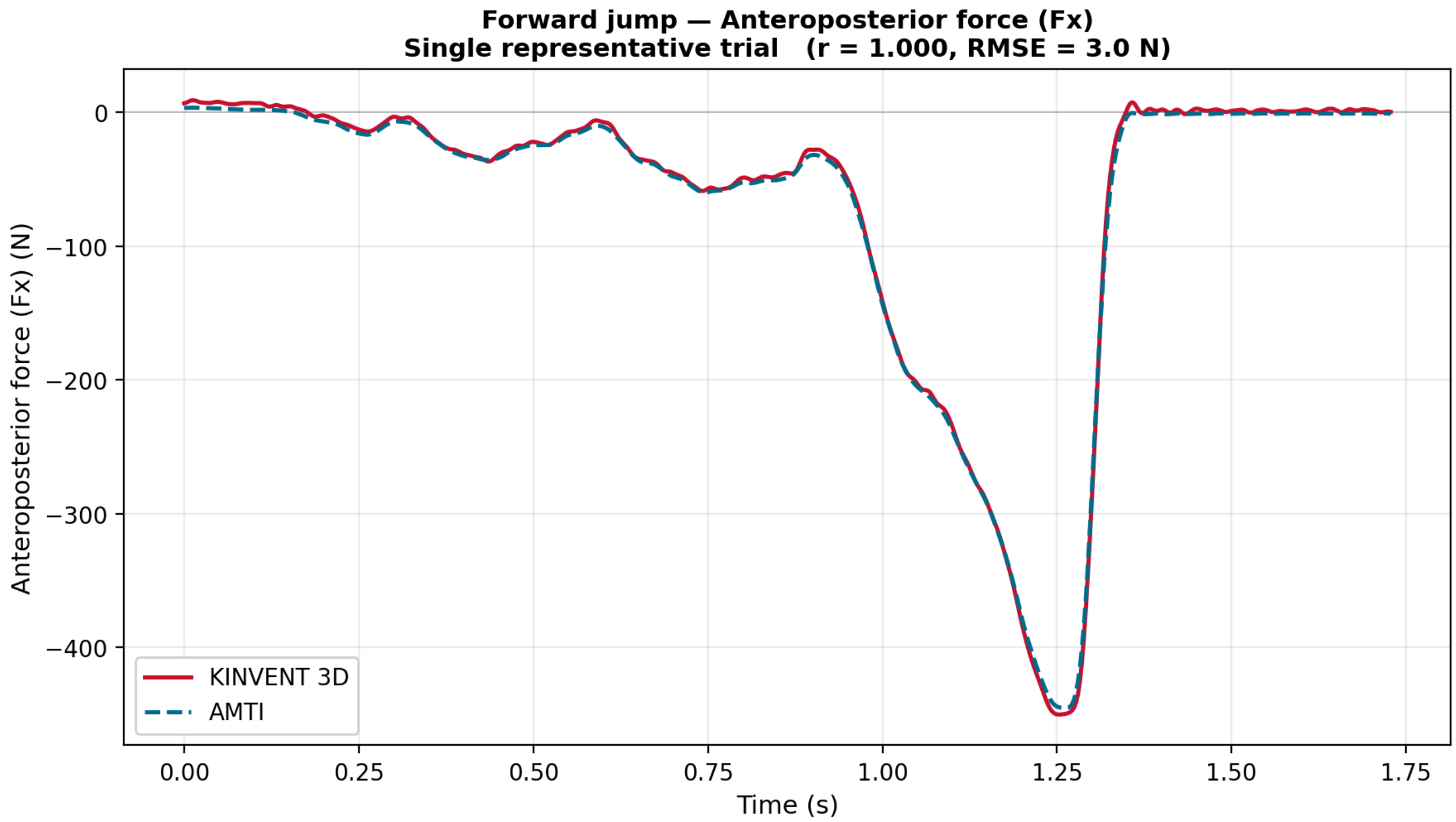

Supplementary Figure 2. Representative anteroposterior ground reaction force curves (Fx) recorded during a forward standing long jump. The root-mean-square error (RMSE) for this trial is displayed above the graph. AMTI: AMTI HPS464508 force-plate (Advanced Mechanical Technology Inc., Watertown, MA, USA); KINVENT 3D: KINVENT 3D-Deltas portable dual force-plate system (KINVENT Biomécanique, Montpellier, France).

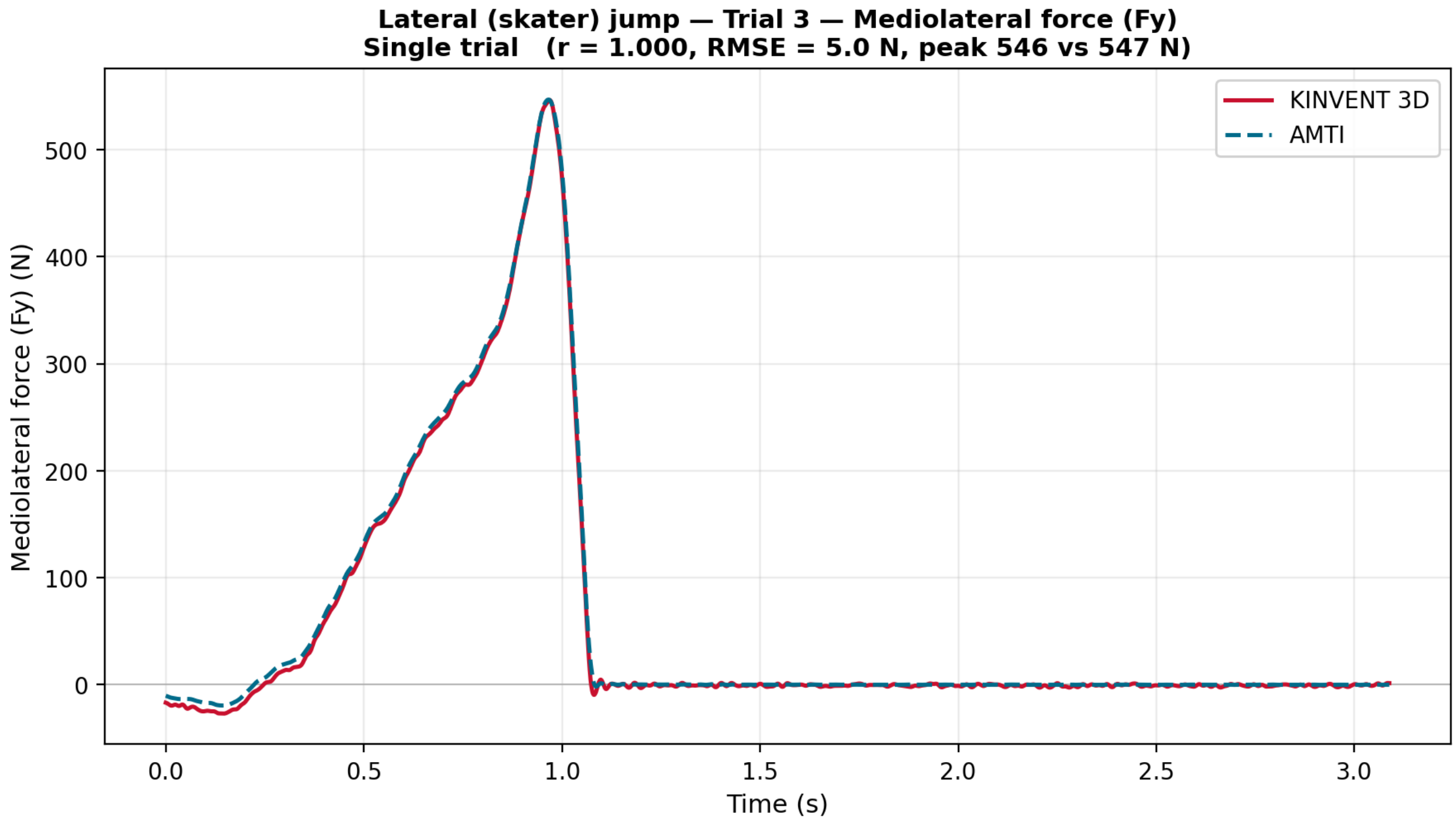

Supplementary Figure 3. Representative mediolateral ground reaction force curves (Fy) recorded during a lateral single-leg sideways jump (“skater” jump). The root-mean-square error (RMSE) and peak values recorded by each force-plate for this trial are displayed above the graph. AMTI: AMTI HPS464508 force-plate (Advanced Mechanical Technology Inc., Watertown, MA, USA); KINVENT 3D: KINVENT 3D-Deltas portable dual force-plate system (KINVENT Biomécanique, Montpellier, France).

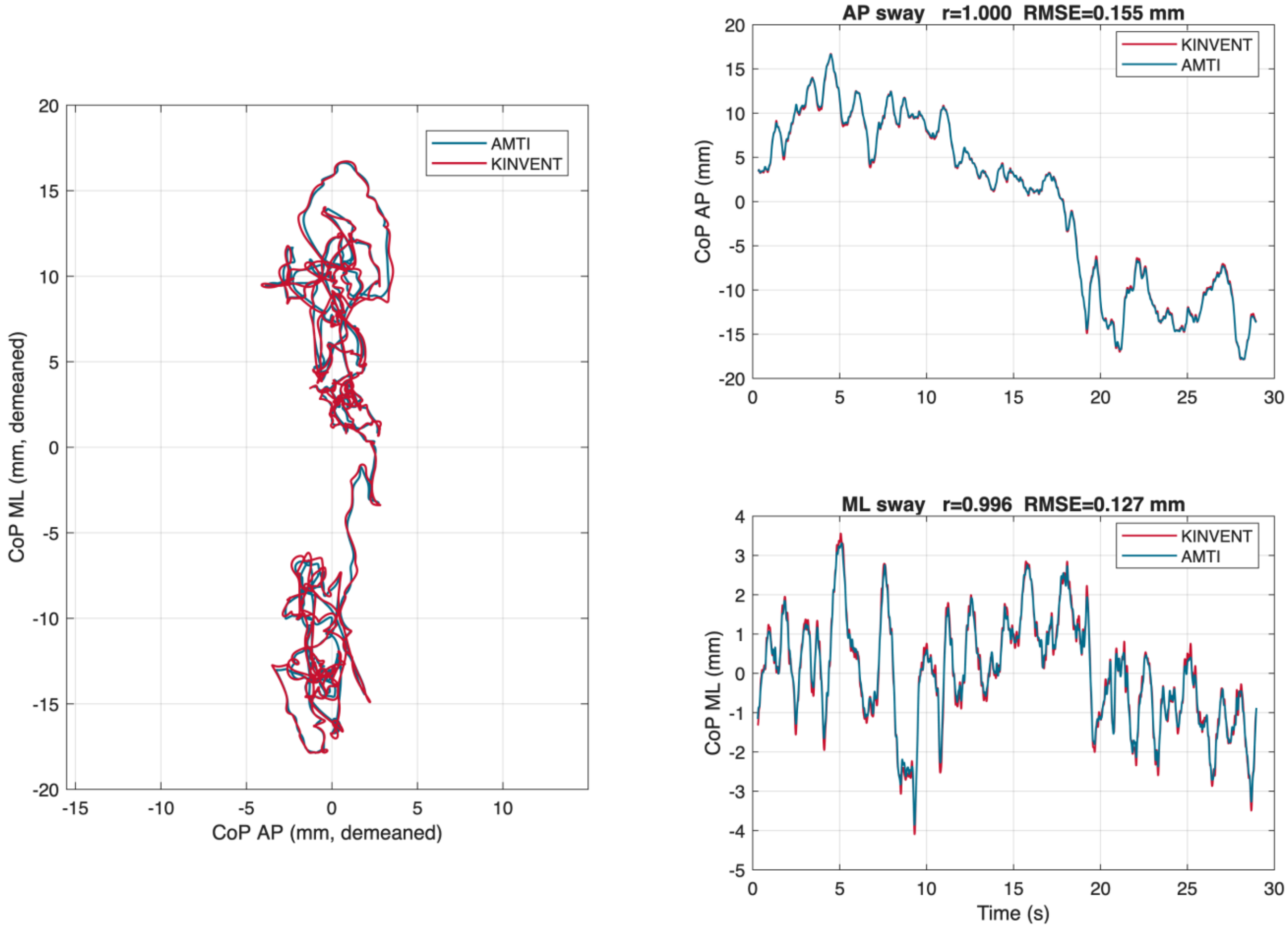

Supplementary Figure 4. Left panel: Representative center of pressure (CoP) displacement recorded during a 30-s quiet-stance static balance test. Right panel: The root-mean-square error (RMSE) values for the anteroposterior (AP) and the mediolateral (ML) CoP sway during this trial are displayed above their respective graphs. AMTI: AMTI HPS464508 force-plate (Advanced Mechanical Technology Inc., Watertown, MA, USA); KINVENT 3D: KINVENT 3D-Deltas portable dual force-plate system (KINVENT Biomécanique, Montpellier, France).
